# Glioma-BioDP: database for visualization of molecular profiles to improve prognosis of brain cancer

**DOI:** 10.1186/s12920-023-01593-w

**Published:** 2023-07-15

**Authors:** Xiang Deng, Shaoli Das, Harpreet Kaur, Evan Wilson, Kevin Camphausen, Uma Shankavaram

**Affiliations:** 1 Radiation Oncology Branch, Center for Cancer Research, National Cancer Institute, National Institutes of Health, 10 Center Drive, Building 10, CRC, Rm B2-3561, Bethesda, MD 20892 USA; 2 Genetics Branch, Center for Cancer Research, National Cancer Institute, National Institutes of Health, Bethesda, MD 20892 USA

**Keywords:** Brain cancer, Glioma, Database, Multi-omics

## Abstract

Cancer researchers often seek user-friendly interactive tools for validation, exploration, analysis, and visualization of molecular profiles in cancer patient samples. To aid researchers working on the both low- and high-grade gliomas, we developed Glioma-BioDP, a web tool for exploration and visualization of RNA and protein expression profiles of interest in these tumor types. Glioma-BioDP is user friendly application that include expression data from both the low- and high-grade glioma patient samples from The Cancer Genome Atlas and enabled querying by mRNA, microRNA, and protein level expression data from Illumina HiSeq and RPPA platforms respectively. Glioma-BioDP provides advance query interface and enables users to explore the association of genes, proteins, and miRNA expression with molecular and/or histological subtypes of gliomas, surgical resection status and survival. The prognostic significance and visualization of the selected expression profiles can be explored using interactive utilities provided. This tool may also enable validation and generation of new hypotheses of novel therapies impacting gliomas that aid in personalization of treatment for optimum outcomes.

**Supplementary Information:**

The online version contains supplementary material available at 10.1186/s12920-023-01593-w.

## Background

Gliomas are the most common types of brain cancers originating in the glial cells. Initially the adult diffuse gliomas were classified according to the microscopic resemblance of the tumor cells with the normal glial cells [1]. In 2000, the World Health Organization (WHO) classified diffuse gliomas into the histological subtypes: astrocytic tumors, oligodendrogliomas, and oligoastrocytomas [2]. These were then graded for their degree of malignancy. Oligoastrocytomas and oligodendrogliomas are graded into grade II or III, while astrocytomas are graded into grades II, III and IV, the grade IV being known as glioblastomas (GBM) and the lower grades are referred as lower grade gliomas (LGG) [3]. Current classification of gliomas is based on genomic alterations in addition to the histopathological classification which are indicative of the aggressiveness of the tumor and patient prognosis [4]. Particularly, for lower grade gliomas (stage II, III), the mutation status of the isocitrate dehydrogenase 1 or 2 (*IDH1/2*) genes and codeletion in 1p19q is considered the key factors for molecular subtyping.

The availability of multi-omic cancer patient data from The Cancer Genome Atlas (TCGA) project has provided cancer researchers with unprecedented opportunities to explore and analyze molecular profiles in relation to patient survival, cancer stage, metastatic state, and other clinical factors [5]. However, downloading the bulk data and writing scripts for analysis and visualization of the data is a daunting task for most clinicians. Web tools like cBioPortal addresses these issues by incorporating interactive visualization and exploration of genetic profiles in different cancers [6]. However, cBioPortal offers limited exploration of cancer-subtype specific changes. While the new version of cBioPortal allows to visualize gene expression changes in glioma histological or molecular subtypes using boxplots, it is not possible to stratify samples by clinical parameters to check the difference in survival related to gene expression change in a specified glioma subtype. To aid the cancer researcher working on gliomas we developed Glioma-BioDP, as an extended version of our previously published web tool GBM-BioDP [7]. Glioma-BioDP enables users with enhanced functions to query gene, protein, and miRNA expression profiles related to molecular subtypes, driver gene alteration status, histological subtypes as well as surgical resection status and patient survival.

### Construction and contents

#### Experimental and clinical data

In the current version of Glioma-BioDP, we collected RNA-seq (v2) and miRNA-seq data from TCGA via Genomic Data Commons (GDC) data portal. We normalized the gene expression count data from RNA-seq and miRNA-seq platforms into log counts per million using the R package edgeR [8]. The normalized data was used for generating all the statistics we show for the expression distribution characteristics. The raw RPPA protein expression data were downloaded from (https://tcpaportal.org/tcpa/). We obtained both level 3 and level 4 data. All the protein expression data were processed and analyzed in a similar way. The gene expression heatmaps were generated using the z-score normalized data for each gene across the samples. Samples and genes were clustered using unsupervised hierarchical clustering with Pearson’s correlation coefficient used as distance/similarity metric.

### Database construction

In the back end, we built a MySQL relational database. All the data presented by the Glioma-BioDP portal are retrieved from the in-house database that hosts information on clinical annotation, subtype information, gene, protein, and miRNA expression. The database also stored all the essential metadata, including the gene, miRNA, and sample information. The patient stratification showed in the portal for both TCGA-GBM and TCGA-LGG is based on the annotation reported by Verhaak and colleagues [9].

The Glioma-BioDP is a PHP based web application. The runtime high level architecture is 3-tiered, consistent with our previous released GBM-BioDP [7]. Processing is done in Python (http://www.python.org/) and visualization is developed using R (http://www.r-project.org/). The application is deployed on an Apache HTTP server (http://httpd.apache.org/) at the National Cancer Institute (NCI).

## Utility and discussion

### Modules

**Fig. 1:**
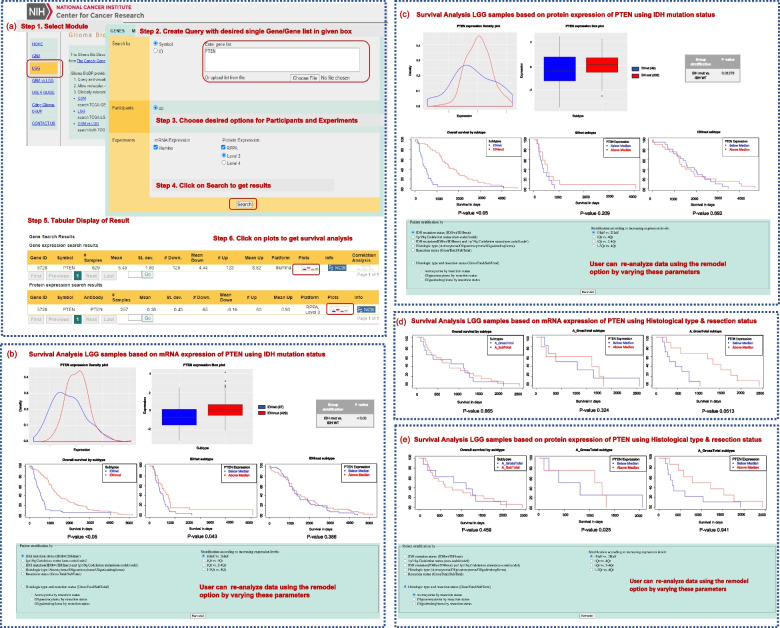
Workflow representing the steps involving the analysis of a gene (*PTEN*) to understand it’s prognostic implication in the LGG using the LGG module of Glioma-BioDP. Panel (**a**): At step 1, the user selects either the LGG, GBM or LGG vs GBM module. At step 2 the user queries for gene (s) of interest followed by in step 3–4, choosing appropriate participants and experiment types for mRNA expression and/or protein expression. Step 5 results in the tabular display of primary results. Next, at step 6, users can click on the plots icon to visualize the survival analysis results for the chosen gene. Panels **b**-**c**: LGG based on IDH mutation status is shown as histogram, boxplot, and survival analysis results for *PTEN* b) mRNA and (**c**) similar results using protein expression respectively. Panel (**d**-**e**): Survival analysis of *PTEN* (**d**) mRNA and (**e**) protein expression in LGG based on histological type and resection status

The Glioma-BioDP webtool has three modules: a) GBM, b) LGG and c) GBM vs LGG, as shown in Fig. 1a. Within each module, sub-modules exist to explore expression profiles and clinical details. Sub-module “GENES” include both mRNA and protein level expression from the TCGA Illumina HiSeq and RPPA platforms respectively. Sub-module “MIRNAs” contain expression data from TCGA Illumina HiSeq platform. The details of modules are described below.

### Core features

1. GBM module: Functionalities and options has been previously described in our publication [7].
2. LGG module: Provide the query and visualization of expression profiles from genes, proteins, or miRNAs from LGG. The visualization panel contains patient stratification with several options as follows:
  - *IDH* wild type (*IDH*wt) vs. *IDH* mutation (*IDH*mut)
  - 1p/19q codeletion status
  - *IDH* and 1p/19q codeletion status
  - histological subtypes including Astrocytoma, Oligoastrocytoma, Oligodendroglioma
  - surgical resection status of gross total or sub-total.
  - Histology and surgical resection for each of the LGG subtypes.

For all the options mentioned above Kaplan-Meier survival plots of expression profiles can be stratified by greater vs. less than mean, and four expression quartiles (1st vs 4th, 1-2 vs 3-4, 1st vs 2-4, 1-3 vs 4th).

The visualization of gene or miRNA expression profiles between any of the above-mentioned stratifications can be visualized with a density plot and a box plot. The *p*-value for the difference of expression levels between these stratifications are calculated using t-tests. Prognostic significance of the gene or miRNA expression in any of the above-mentioned stratification is visualized with Kaplan-Meier survival curves stratified by four expression quartiles as described above.

3) GBM vs LGG module: To explore prognostic significance of gene expression in GBM vs. LGG. Visualization is shown in a side-by-side comparison with Kaplan–Meier survival plots. The patient samples can be stratified by greater vs less than mean, and four expression quartiles of the queried gene or miRNA as described above.

For all three modules heatmaps for multiple query genes are shown to visualize their gene expression clustering with respect to clinically relevant parameters like molecular subtypes and the prognostic index.

### Workflow and applications of Glioma-BioDP

Glioma-BioDP facilitates the user to assess the expression pattern and prognostic potential of desired gene in specific brain tumor, i.e. Glioblastoma (GBM) or Low-grade gliomas (LGGs) or both. Here, we describe the brief workflow of analyses using a clinically relevant gene *PTEN* as an example. The tumor suppressor gene *PTEN* plays important roles in the regulation of cell proliferation, apoptosis, and DNA damage repair [10]. Treatment of *PTEN*-deficient tumors with *PI3K* pathway inhibitors are being investigated for some cancer types [11]. The loss of *PTEN* expression has been indicated to be an early event in glioma, with mutations occurring between 5 and 40% of glioma cases.

To get comprehensive understanding about the role of a selected gene (e.g. *PTEN*) in LGG subtypes from diverse perspective, we have integrated different molecular types such as mRNA, protein expression, molecular and histological subtypes-based stratification of tumors. Complete workflow for the analysis of *PTEN* in molecular and histological subtypes using LGG module is represented in the Fig. 1a. Here, we have selected mRNA and protein expression-based query for the analysis of *PTEN* (steps 1–4). Glioma-BioDP allows the user to select any of desired options provided on the platform. Subsequently, the user will be directed to the tabular display of the gene and protein expression pattern in LGG samples. By clicking on the plots the user will be directed to the graphical interface (see steps 5–6). Figure 1b shows the histogram distribution and box plots of differences *PTEN* mRNA expression in IDH-mut vs. IDH-wt. The 3 survival plots in Fig. 1b shows the difference between IDH-mut vs. IDH-wt (*p*-value < 0.05), within each subset, if there is a difference between greater or less than median expression. Like the mRNA expression-based query, protein expression-based query can be performed to see differences in histogram, boxplots, and survival between different molecular subtypes of LGG (Fig. 1c). Importantly, Glioma-BioDP allows the user to rebuild their survival models employing different parameters based on molecular features: *IDH* mutation and 1p/19q codeletion status, histological subtypes, surgical resection status, varying quartile ranges, etc. The resulting KM plots from the stratification of samples using histological type and resection status for mRNA and protein expression-based queries are shown in Fig. 1d and e respectively.

## Case studies

The Glioma-BioDP tool’s functionality and potential clinical relevance is demonstrated through the analyses of the following genes: *PTEN*, *NES, TERT, MGMT* and *EGFR*. The clinical relevance of *PTEN* in gliomas is explained in the previous section. *NES* is a gene that codes for nestin, an intermediate filament found in vascular endothelial cells that is upregulated in tumors to allow for increased angiogenesis at the tumor site [12]. *TERT* codes for telomerase reverse transcriptase, a protein key to the maintenance of telomeres and one whose expression is upregulated in a subset of gliomas through promoter mutation or by other means to facilitate tumor progression [13]. *MGMT* promoter methylation and subsequent *MGMT* gene inactivation is common in malignant gliomas. Epigenetic *MGMT* promoter methylation has been shown to be associated with better clinical outcomes for patients treated with temozolomide (TMZ) and radiotherapy due to a decrease in tumor DNA repair [14]. This observation has great clinical relevance in terms of patient selection for chemo and radiotherapy. *EGFR* amplification and mutation are a signature genetic abnormality in GBM [15] and may be explored as a therapeutic target. Using these examples, we described the prognostic significance of each of these genes in LGG and GBM.

### Prognostic value of the expression of *PTEN* in LGG subtypes

**Fig. 2:**
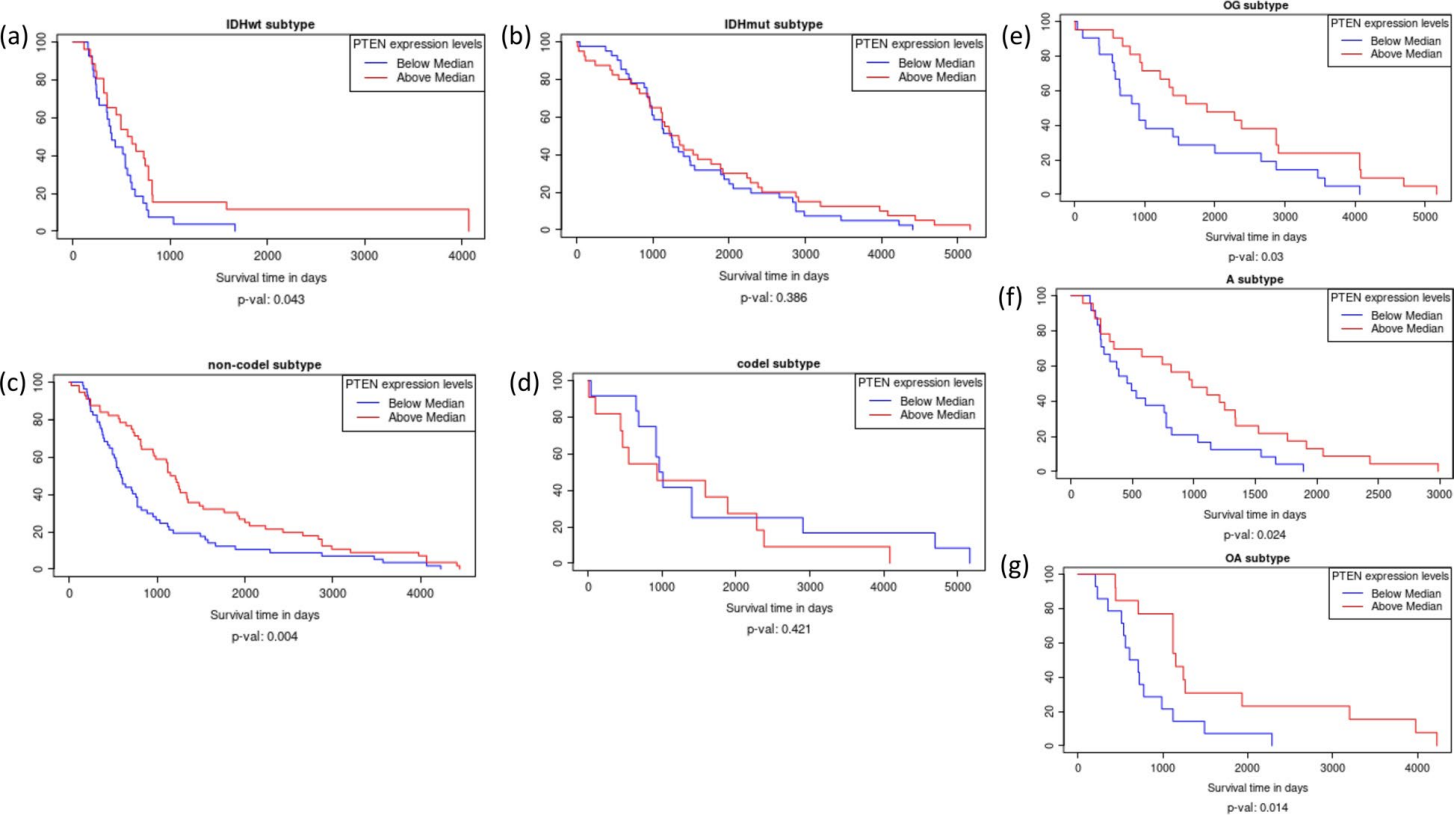
**a** *PTEN* expression related to survival in *IDH*wt compared to *IDH*mut LGG. **b** *PTEN* expression related to survival in 1p19q non-co-deleted compared to 1p19q co-deleted LGG. **d** *PTEN* expression related to survival in oligodendroglioma. **d** *PTEN* expression related to survival in astrocytoma. **e** *PTEN* expression related to survival in oligoastrocytoma

Expanding on the *PTEN* gene search in LGG module, the potential clinical relevance of overexpression of *PTEN* mRNA level was shown to be associated with better survival in *IDH*wt but not in *IDH*mut LGGs (Fig. 2a, b, Kaplan–Meier analysis, *p*-value = 0.043 and 0.386 respectively, samples stratified by > median or < median of *PTEN* expression). Also, querying *PTEN* expression in 1p19q co-deleted vs non-co-deleted LGGs showed that *PTEN* overexpression is associated with better survival in 1p19q non-co-deleted LGGs but not in 1p19q co-deleted LGGs (Fig. 2c, d, Kaplan–Meier analysis, *p*-value = 0.004 and 0.421 respectively, samples stratified by > median or < median of *PTEN* expression). Further, querying for histological subtypes of LGG, it was seen that *PTEN* overexpression was associated with better survival in all histological subtypes: oligodendroglioma, astrocytoma, and oligoastrocytoma (Fig. 2e, g, Kaplan–Meier analysis, *p*-value = 0.03, 0.024 and 0.014 respectively, samples stratified by > median or < median of *PTEN* expression). There are investigations underway for finding treatment strategies for targeting *PTEN*-deficient cancers. Association of *PTEN* overexpression with improved survival in glioma subtypes with poor prognosis (*IDH*wt and 1p19q non-co-deleted) may provide rationale for investigating the effects of these therapies in these glioma subtypes.

### Prognostic value of the expression of genes *NES*, *TERT* and *MGMT* in LGG vs GBM

**Fig. 3:**
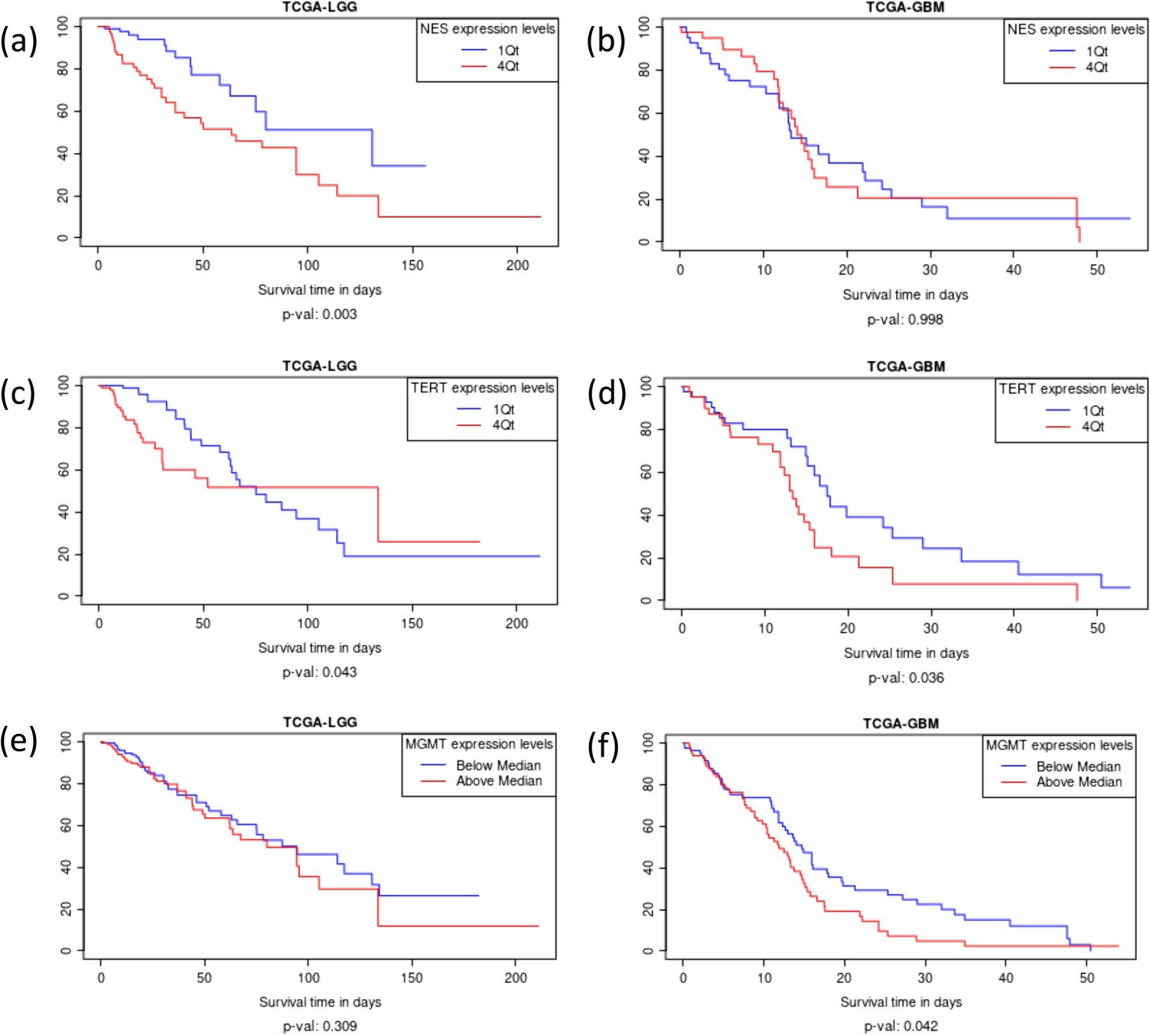
Prognostic significance of genes *NES, TERT* and *MGMT* in LGG vs GBM. **a** *NES* protein expression comparison related to survival in GBM vs LGG. **b** *TERT* protein expression comparison related to survival in GBM vs LGG. **c** *MGMT* protein expression comparison related to survival in GBM vs LGG

The webtool can showcase genes that are manipulated either LGG or GBM or equally in the GBM and LGG tumor microenvironments. *NES* is an example of a gene that elucidates the Glioma-BioDP webtool’s ability to identify genes that have a significant effect on prognosis in LGG but not GBM. From Fig. 3a, b, elevated *NES* expression levels are shown to be associated with decreased survival times in LGG but not GBM (*p*-value: 0.003 vs. *p*-value: 0.998, 1^st^ vs. 4^th^ Quarter analysis).

*TERT* is an example of the genes that have prognostic significance in both LGG and GBM. *TERT* expression levels are shown in Fig. 3c, d to have a similar effect on patient survival times in both GBM and LGG (*p*-value: 0.036 vs *p*-value: 0.043, 1^st^ vs 4^th^ Quarter analysis).

*MGMT* shows the webtool’s ability to highlight genes that show profound impact and significance in glioblastoma (GBM) but not low-grade glioma (LGG). The beneficial effect of *MGMT* promoter methylation and gene inactivation is corroborated by Glioma-BioDP in Fig. 3e, f, showing that decreased *MGMT* protein expression is associated with longer survival times in the clinical setting as the tumor progresses (*p*-value; 0.309 in LGG vs *p*-value: 0.042 in GBM, below and above median analysis).

### Prognostic value of the expression of *EGFR* in molecular subtypes of LGG and GBM

**Fig. 4:**
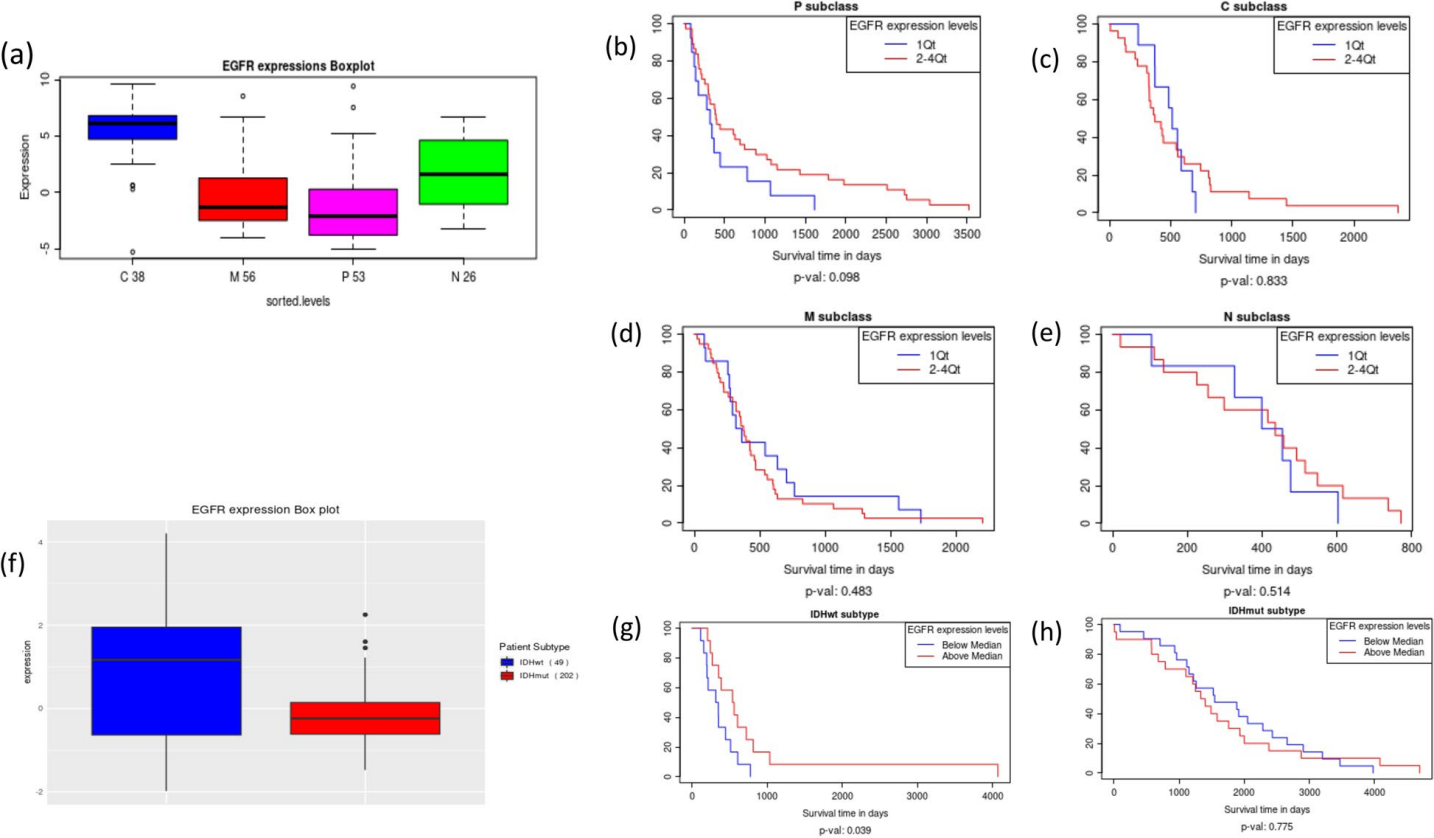
**a** *EGFR* mRNA expression in GBM molecular subtypes. Classical vs other subtypes *p*-value < 0.001 **b**-**e** *EGFR* mRNA expression related to survival in GBM molecular subtypes: (**b**) pro-neural (**c**) classical (**d**) mesenchymal (**e**) neural. **f** *EGFR* protein expression in LGG subtype *IDH*wt compared to *IDH*mut. *P*-value < 0.001 (**g**) *EGFR* protein expression related to survival in *IDH*wt subtype of LGG. **h** *EGFR* protein expression related to survival in *IDH*mut subtype of LGG

To explore the prognostic effects of *EGFR* expression in molecular subtypes of LGG and GBM, we queried Glioma-BioDP. When we queried for *EGFR* expression in GBM subtypes on the GBM module, it could be observed that the mRNA and protein expression of *EGFR* is significantly higher in the classical subtype of GBM compared to all other subtypes (Fig. 4a shows mRNA expression boxplot, classical vs other subtypes *p*-value < 0.001). Though patient stratification by *EGFR* expression within each molecular sub-type do not show significant association with survival, in the proneural subtype strong trend is seen for association of better survival with overexpression of *EGFR* (Fig. 4b, Kaplan–Meier analysis, *p*-value = 0.098, sample stratification by 1st quartile vs 2–4 quartiles). Patient survival in the other molecular subtypes of GBM did not show any association with *EGFR* expression level (Fig. 4c-e). On the other hand, in LGG subtypes stratified by the presence of *IDH* mutation (wild type *IDH* or *IDH*wt vs mutated *IDH* or *IDH*mut), it could be seen that *EGFR* mRNA (TCGA RNA-Seq data) and protein (TCGA RPPA data) expression is significantly higher in *IDH*wt samples compared to *IDH*mut samples, as visualized using density plots and box plots for protein expression (Fig. 4f, *p*-value < 0.001). From the Kaplan–Meier survival plots, it could be seen that overexpression of *EGFR* protein level is associated with better survival in *IDH*wt (Fig. 4g), but not in the *IDH*mut subtype of LGG.

Thus, the functionality of Glioma-BioDP is evident through the juxtaposition of these genes whose effect on patient outcomes is widely different yet similarly potent in either GBM or LGG or both settings. Also, querying Glioma-BioDP enables users to explore the mRNA and protein level profiles for their genes of interest in context of the molecular and histological subtypes.

## Conclusions

In the age of big data in cancer genomics, there is an opportunity for cancer researchers to use and explore the patient genomic data from large tumor cohorts such as TCGA, to improve their understanding of genomic correlates to patient prognosis. However, there is a need for availability of the data in easy to explore format and intuitive visualization that would enable the cancer researchers to make use of that enormous data. Glioma-BioDP as a user-friendly web tool offers intuitive visualization and query of gene and miRNA expression data in gliomas in context of specific histological and molecular subtypes of these tumors. In addition to our previously published tool GBM-BioDP, the new tool Glioma-BioDP enables exploration of the prognostic significance of transcriptomic and proteomic features from low grade to high grade gliomas in subtype-specific manner.

In comparison to a previously published tool GliomaDB [16], our tool Glioma-BioDP offers more intuitive and useful visualizations by enabling the users to look at gene or miRNA expressions between different histological, as well as molecular subtypes of gliomas. As described in our case studies, with Glioma-BioDP users get useful information on the survival of the glioma patients depending on the queried gene expression in context of the histological and/or molecular subtypes of gliomas. Including this information is a critical feature of Glioma-BioDP as the subtypes are linked to varied degree of patient prognosis in glioma, and the expressions of different genes may have different implications in prognosis depending on the glioma subtype. An example of this functionality is described by the association of *PTEN* overexpression with better survival in *IDH*wt and 1p19q non-co-deleted LGGs. Another example is that *EGFR* gene and protein level expressions are associated with prognosis in the glioblastomas (grade IV glioma), and even in the lower grade gliomas (grade II-III), *EGFR* protein level expression is associated better prognosis with the *IDH*wt molecular subtype which is the high-risk subtype compared to *IDH*mut.

In upcoming version of this tool, we are integrating miRNA and protein expression data into the LGG and LGG vs GBM modules. In the near future we are looking forward to incorporate data from more omic platforms like mutation, copy number and DNA methylation that would expand the usability of this tool.

### Supplementary Information

**Additional file1.**

**Additional file 2.**

## Data Availability

**Availability:** Glioma-BioDP web tool with user manual is available from: https://glioma-biodp.nci.nih.gov Contact: uma@mail.nih.gov.
