# Supplementary material for: Glioma-BioDP: database for visualization of molecular profiles to improve prognosis of brain cancer
Source: BMC Med Genomics. 2023 Jul 15;16:168. doi: 10.1186/s12920-023-01593-w (PMC10350252; doi:10.1186/s12920-023-01593-w)
Supplement: Supplementary file 1 — Additional file1. [file 12920_2023_1593_MOESM1_ESM.pdf]

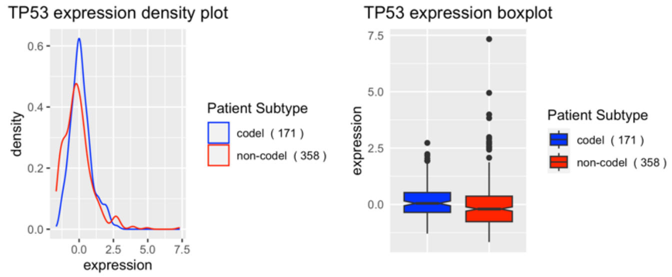

stratification	pvalue	CI95low	CI95high	mean_non_codel	mean_codel
non-codel-codel	0.00026	0.046	0.368	0.14	-0.067

Additional Figure 1 for review: Changes implemented in the upcoming version of Glioma-BioDP with gene expression notched boxplots and results table showing mean and 95% confidence interval of the groups being compared. Examples shows TP53 gene expression in LGG subtypes 1p19q codel vs 1p19q non-codel.

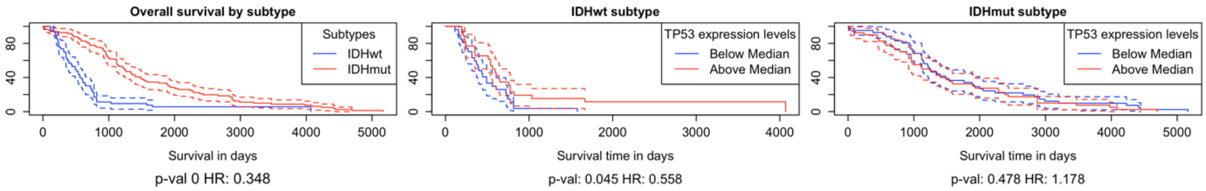

Additional Figure 2 for review: Changes implemented in the upcoming version of Glioma-BioDP with HR and 95% CI added to the survival plots. Example shows survival in patients with TP53 gene up/downregulation in LGG subtypes IDH mut vs non-mut.
